# Peptidases Compartmentalized to the *Ascaris suum* Intestinal Lumen and Apical Intestinal Membrane

**DOI:** 10.1371/journal.pntd.0003375

**Published:** 2015-01-08

**Authors:** Douglas P. Jasmer, Bruce A. Rosa, Makedonka Mitreva

**Affiliations:** 1 Department of Veterinary Microbiology and Pathology, Washington State University, Pullman, Washington, United States of America; 2 The Genome Institute, Washington University School of Medicine, St. Louis, Missouri, United States of America; 3 Department of Medicine and Department of Genetics, Washington University School of Medicine, St. Louis, Missouri, United States of America; McGill University, Canada

## Abstract

The nematode intestine is a tissue of interest for developing new methods of therapy and control of parasitic nematodes. However, biological details of intestinal cell functions remain obscure, as do the proteins and molecular functions located on the apical intestinal membrane (AIM), and within the intestinal lumen (IL) of nematodes. Accordingly, methods were developed to gain a comprehensive identification of peptidases that function in the intestinal tract of adult female *Ascaris suum*. Peptidase activity was detected in multiple fractions of the *A. suum* intestine under pH conditions ranging from 5.0 to 8.0. Peptidase class inhibitors were used to characterize these activities. The fractions included whole lysates, membrane enriched fractions, and physiological- and 4 molar urea-perfusates of the intestinal lumen. Concanavalin A (ConA) was confirmed to bind to the AIM, and intestinal proteins affinity isolated on ConA-beads were compared to proteins from membrane and perfusate fractions by mass spectrometry. Twenty-nine predicted peptidases were identified including aspartic, cysteine, and serine peptidases, and an unexpectedly high number (16) of metallopeptidases. Many of these proteins co-localized to multiple fractions, providing independent support for localization to specific intestinal compartments, including the IL and AIM. This unique perfusion model produced the most comprehensive view of likely digestive peptidases that function in these intestinal compartments of *A. suum*, or any nematode. This model offers a means to directly determine functions of these proteins in the *A. suum* intestine and, more generally, deduce the wide array functions that exist in these cellular compartments of the nematode intestine.

## Author Summary

Past research has demonstrated that the nematode intestine has value for developing new methods of therapy and control of parasitic nematodes, as related to both vaccines and other anthelmintics. Yet, information related to basic intestinal cell biology is very limited. Research progress reported here moves towards the comprehensive identification of proteins (peptidases and others), and hence functions, that are sited on the apical intestinal membrane and within the intestinal lumen of adult female *Ascaris suum*. These advances provide an unprecedented research model to determine critical functions sited at these locations and to develop approaches to inhibit those functions. Comparative analysis among diverse parasitic species raises expectations that the results from *A. suum* can be applied to many parasitic nematodes for which similar research is technically impossible to perform.

## Introduction

Parasitic nematodes cause major diseases of humans, directly affecting more than two billion people on a global scale [1]. Diseases they cause in food animal species also pose significant constraints to agricultural production and thus, indirectly impact human health in regions of the world where nutritional resources are limited. Acquisition of resistance to contemporary anthelmintics by many species of parasitic nematodes is on the rise [2], [3]. Hence, the need for research to identify new targets for therapies to treat and control infections by these pathogens has never been greater. The nematode intestine is one tissue of importance in this context.

The intestine of parasitic nematodes is formed by a single cell layer. The apical intestinal membrane (AIM) forms an intestinal lumen (IL), and both the AIM and IL are accessible from the outside host environment. In combination, these two intestinal cell compartments form a major parasite interface with the host that performs a wide range of biochemical and cellular functions essential for survival of these pathogens. Consequently, knowledge of functions sited at this interface, and cellular processes needed to maintain it, could lead to approaches that disrupt intestinal cell functions critical for parasite survival.

Research on the AIM of the parasitic nematode *Haemonchus contortus*, a gastrointestinal nematode of small ruminants, has demonstrated the importance of AIM glycoproteins as targets for vaccines [4]–[6], advances on which have been extended to hookworms [7], and in stimulating host mucosal immune responses during an infection [8]. These advances have underscored the unique value of the nematode intestine as a target for vaccines. Importance of the *H. contortus* intestine as a drug target was demonstrated relative to a benzimidazole anthelmintic [9], which when used to inhibit AIM biogenesis caused catastrophic damage to the *H. contortus* intestine. The nematode AIM is also a primary target for crystal protein toxins produced by *Bacillus* spp., which have efficacy against multiple parasitic nematodes [10]. Properties unique to the nematode intestine appear to account for anthelmintic effects of both the benzimidazole and crystal protein treatments. Consequently, the nematode intestine presents multiple biological characteristics that can be targeted by new therapies for treatment and control of parasitic nematodes.

Nevertheless, details of intestinal cell functions remain obscure, in part due to research challenges imposed by the small size of many parasitic nematodes, including *H. contortus*. In this context, the size of adult *Ascaris suum*, the large round worm of swine (20–35 cm compared to ca. 2.5 cm for adult *H. controtus*), offers research capabilities absent with many other nematode species. The close relationship to *A. lumbricoides*, the most globally prevalent parasitic helminth of humans [11], makes *A. suum* research particularly relevant to this human pathogen and global health.

Previous comparisons of transcripts expressed in the intestines of *H. contortus*, *Caenorhabditis elegans* and *A. suum* identified likely, orthologous intestinal proteins in common among these species, which represent a phylogenetic distance spanning an estimated 350 million years [12]. These similarities were detected despite the utilization of markedly different nutrient sources by these species, e.g. host blood, *H. contortus*; bacteria, *C. elegans*; and host intestinal content, *A. suum*. This comparative approach, coupled with larger genome projects [13], has begun to clarify orthologous protein groups with potential for broad biological application to many species of nematodes, whether parasitic or not.

In research reported here, we coupled advantages conferred by the large size of *A. suum*, and past progress made with *H. contortus*, to develop an improved model for investigating functions located on the AIM and in the IL of *A. suum*. Previous research clarified properties of some glycoproteins and peptidases located on the *H. contortus* AIM surface [4], [6], [14], [15], and intestinal transcript analysis identified candidate, orthologous intestinal proteins from *A. suum* [12], which we hypothesize perform related functions in these two species. In addition to peptidases, many other proteins are expected to reside at the nematode AIM, or in the IL, fulfilling roles in digestion and nutrient transport, as examples. The approach reported here facilitated identification of an extensive set of apparent *A. suum* peptidases that are sited at the AIM and IL, in addition to many other putative AIM and IL proteins. The results have greatly expanded concepts on apparent functions that reside at the *A. suum* AIM and in the IL. The approach also established *A. suum* as a unique model to directly investigate i) functions that are located in these nematode intestinal cell compartments and ii) methods to inhibit those functions, in context of improved or novel therapies and methods of parasite control.

## Methods

Additional details are provided in Supporting Information (S1 Text).

### Parasite material and intestinal fractionation

Adult female *Ascaris suum* were obtained from swine infected as weanling pigs (mixed breed, Swine Center, Washington State University) 60 to 70 days post-infection. Infections were initiated with *A. suum* eggs collected from uteri of three or more patent female worms. Eggs were cultured in distilled water at room temperature for 30 days to allow eggs to embryonate, and then treated with 0.25% sodium hypochlorite for up to seven minutes to decoat the eggs, followed by three rounds of washes in 50 ml distilled water, and then stored at 5C in the refrigerator until use. Parasite intestinal samples were dissected from two or more freshly isolated worms maintained in ice cold phosphate buffered saline (PBS, pH 7.4). Intestinal samples were stored −80C until used. Intestinal tissue samples used for centrifugal fractionation and Concanavalin A (ConA) isolation and analysis were not necessarily from the same worm preparations.

### Protein fractions

#### Whole intestine and pseudocoelomic fluid

To generate protein samples for peptidase experiments, frozen intestinal samples were ground in liquid nitrogen using a mortar and pestle. Approximately 50 µl of ground frozen sample were transferred to a microfuge tube and 1 ml of PBS was added to the sample which was briefly vortexed and then treated in three rounds of a freeze (−20C) thaw (4C) cycle. The homogenate was then centrifuged at 5,000×*g* for 10 minutes, which produced a pellet (P1) and supernatant (S1). The S1 supernatant was centrifuged at 50,000×*g* for 30 minutes producing a 5,000 to 50,000×*g* pellet (P2) and supernatant (S2). Pseudocoelomic fluid (PF) was obtained by making an incision with iris scissors along the body wall located between the lateral attachments of the intestine to the body wall. This method avoided damage to the intestine and allowed access to pseudocoelomic fluid, which was collected with a needle and syringe from the body cavity.

#### Cannulation and collection of intestinal perfusates

Adult *A. suum* worms were collected from infected pigs and immediately placed in 37C PBS. They were processed within three hours for cannulation and collection of perfusates. The anterior ends of adult female *A. suum* worms were removed by scalpel just below the esophagus. A blunt needle cannula (25 gauge), blunted by grinding with a Dremel grinding wheel, was inserted into the anterior end of the intestine. Superglue gel was applied around the cannula, about three quarters of the way up the cannula prior to insertion. The cannula was inserted until worm tissue came into contact with the superglue. Liquid superglue was then applied around the junction where the anterior end of the worm encountered the superglue gel. The posterior one sixth of the worm was then removed to eliminate a fragile section of the intestine. Approximately one centimeter of the remaining posterior end of the body was resected to expose the intestine. Perfusates injected into the anterior end of the intestine were collected from the posterior end that was placed onto Parafilm M (Pechiney Plastic Packaging Co., Chicago, IL), which allowed manipulation of the posterior end of the intestine in positions to reduce contamination of pseudocoelomic fluid (PF). Perfusion with dye indicated a lumenal content of ca. 50 µl. Cannulated intestines were perfused with approximately 300 to 500 µl of PBS (PBS perfusate), followed by a similar volume of 4 M urea in PBS (4MU perfusate), each delivered with a tuberculin syringe attached to the cannula. Maintenance of worms at 37C prior to cannulation appeared to enhance the flow of perfusates through the intestine. Perfusate samples were stored at −20C until used in various experiments. Protein concentrations for all samples were determined using a bicinchoninic acid assay (Micro BCA Protein Assay Kit, Thermo Scientific, Rockford, IL). The PBS and 4MU perfusates were collected as a paired set from individual worms. Results from peptidase assays and ConA blots were presented for a set of perfusates. A paired set of perfusates was also used for analysis by mass spectrometry. Perfusate samples were derived from different worms used to generate intestinal tissue samples for other experiments involving whole tissue or fractions from whole intestinal tissue.

#### Concanavalin A (ConA) binding proteins

Whole intestinal lysate was prepared with peptidase inhibitors (1 µM Pepstatin A, 1 mM PMSF, 10 mM 1,10 Phenanthroline, 5 mM Iodoacetamide, Sigma, St. Louis, MO) and solubilized with 1% sodium dodecyl sulfate (SDS). The lysate was clarified by centrifugation at 5,000×g and then the supernatant was diluted to 0.25% SDS with Binding Buffer containing divalent cations (50 mM TRIS [pH 7.4], 500 mM NaCl, 1 mM each MgCl_2_, MnCl_2_, and CaCl_2_). ConA-agarose beads (Sigma, St. Louis, MO) were incubated with solubilized lysate (2 mg per 200 µl packed beads). The mixture was incubated with inversion for two hours, and then washed three times with nine bead volumes of Binding Buffer (50 mM TRIS, pH 7.4, 500 mM NaCl), then 300 µl washes of potassium thiocyanate (0.25 M, 0.5 M, 1 M and 2 M) in 20 mM TRIS [pH 7.4], 0.2% Triton X-100, two washes per concentration), and then two Binding Buffer washes (9 bead volumes each). Proteins remaining on the beads were eluted by boiling in 0.1% SDS for separation by SDS-polyacrylamide gel electrophoresis (PAGE) and mass spectrometric analysis.

### Histological analysis

10% formalin fixed adult female worms embedded in paraffin (Histology Laboratory, Washington State University) were sectioned, attached to glass slides and deparaffinized and steam treated. To assess specificity of ConA binding, sections were treated with sodium periodate (5 mM in 50 mM sodium acetate buffer, pH 4.5), followed by sodium borohydride (50 mM in PBS, pH 7.4) to disrupt carbohydrates containing vicinyl hydroxyl groups. Slides were ten treated with 0.3% hydrogen peroxide in methanol for 30 min at 25°C to eliminate endogenous peroxidases and then incubated with ConA-horse radish peroxidase (HRPO). Binding was localized by development with Metal Enhanced DAB Substrate (Thermo Scientific, Rockford, IL.). Sections were then counterstained with Mayer's haematoxylin (Thermo Scientific, Fremont, CA).

### SDS-PAGE analysis

Protein samples were separated by SDS-PAGE under non-reducing conditions, using 17% to 7% polyacrylamide gradient gels using previously described methods [15]. Proteins were either stained with Coomassie Brilliant Blue R-250 or transferred to nitrocellulose filters. Filters were then incubated with ConA-HRPO to localize glycoproteins by chemiluminescence (Pierce ECL Western Blotting Substrate, Thermo Scientific, Rockford IL) recorded on x-ray film (Kodak O-MAT). To assess specificity of ConA binding, replicate nitrocellulose filters were treated with sodium periodate as described for tissue sections, and control filters were treated the same, but excluding sodium periodate.

### Peptidase assays

For analysis of soluble protein, samples were adjusted to 1% TX-100 and used in assays at 0.5 to 4 µgs (depending on assay) in each well. Buffers used were 100 mM citrate phosphate (pH 5.0–7.0) and 100 mM phosphate (pH 8.0). Samples were incubated with Bodipy FL casein (E6638, Life Technologies, Grand Island, NY) for two hours in a C-1000 Touch thermal cycler with a CFX96 Optical Reaction Module (Bio-Rad, Hercules, CA) at 37°C, in a total volume of 50 µl. Assays were conducted in triplicate using 96 well PCR plates (Bio-Rad, Hercules, CA). Fluorescence signal was measured (excitation 490 nm; emission 530 nm). Net fluorescence signal was determined by subtraction of starting values from end values. Mean fluorescence was calculated for no protein controls after incubation with Bodipy FL casein at a given pH for two hrs. This background value was subtracted from each fluorescence value obtained for samples with protein. Activity was expressed as mean relative fluorescence units per µg of protein.

For analysis of ConA binding proteins, intestinal supernatant S1 solubilized in 1% TX-100 was incubated with beads (1 mg per 100 µl packed beads) for 2 hours with inversion, then washed with Binding Buffer containing divalent cations (50 mM TRIS, [pH 7.4], 500 mM NaCl, 1 mM each MgCl_2_, MnCl_2_, and CaCl_2_). ConA-agarose beads with bound proteins (10 µl) were transferred to wells of 96 well flat-bottomed plates (Corning Costar, Corning, NY) in 50 µl total volume for assays as described for peptidase assays. Beads with no proteins were used for no protein controls. The 96 well plates were rotated during incubation (50 rpm) to ensure mixing. Reaction supernatants from which beads were eliminated were transferred to a 96-well PCR plate (Bio-Rad, Hercules, CA) for fluorimetric measurements, as described for peptidase assays. Alternatively, SDS (1.0%) solubilized intestinal lysates were used for ConA bead isolation and analysis by mass spectrometry and testing in peptidase assays, but produced no detectable activity.

### Statistical analysis

Mean fluorescence units generated from treatments in peptidase assays were compared first by analysis of variance (ANOVA), followed by Tukey's multiple comparison of means. ANOVA was conducted among treatment groups for individual pHs. For inhibition experiments, ANOVA and multiple means comparisons were conducted to identify means of inhibitor treated groups that differed from the untreated control group at a given pH. For significance with ConA-bead isolated proteins, mean fluorescence at each pH tested was assessed by 95% confidence intervals to determine if the intervals were above zero.

### Mass spectrometry – sample preparation

ConA bead-isolated proteins were separated on SDS-PAGE gels in two lanes, one of which was Coomassie Blue stained and the second was transferred to nitrocellulose and probed with Con A-HRPO. Bands detected in blots were used to align with stained bands in the gel, which were excised and then prepared for *in situ* trypsin digestion and analysis by LC-MS/MS as described [8]. Proteins in PBS and 4MU perfusates, PF, and P2 pellets were precipitated with the “2-D Clean-up kit” (GE Healthcare Life Sciences), and then resolubilized in 8M urea, 100 mM Tris, pH 8.5 (20 µl) for 30 min at 37°C. Samples were then reduced with 1 mM tris(2-carboxyethyl)phosphine TCEP (2 µl of 10 mM TCEP stock) at room temperature for 30 minutes, followed by alkylation with 20 mM Iodoacetamide for 30 minutes in the dark. Then, samples were quenched with 10 mM DTT for 15 minutes and diluted 1∶4 dilution in Tris (pH 8.5).

### Liquid chromatography, tandem mass spectrometry (LC-MS/MS)

Proteins were digested sequentially with endoprotease Lys-C (cleaving lysine at the C-terminus) and trypsin as previously described [16] and then processed for liquid chromatograpy-tandem mass spectrometry (Supplemental Materials).

### Mass spectrometry data processing and analysis

LC-MS data files (MS2 centroided) were used for database searching with MASCOT (Matrix Science, version 2.3.0.0) using previously described software settings [17], against the deduced *A. suum* proteome [18] and the *Sus scrofa* proteome (Uniprot, downloaded Sept. 2012) to identify potential host contamination. Thresholds for detection were set in accordance to the suggestions by the Scaffold documentation, and as described in other recent proteomics studies [19]–[21]. Proteins that contained similar peptides and could not be differentiated based on MS/MS analysis alone were grouped to satisfy the principles of parsimony.

### Ethics statement

The research involving use of swine was reviewed and approved by the Washington State University Institutional Animal Care and Use Committee, protocol #04097-004., approved on 12/19/2013. Guidelines are provided by the Federal Animal Welfare Act, USA.

## Results

### *A. suum* homologues of previously reported, broadly conserved, intestinal peptidases

Peptidases from four different major classes (Aspartic, cysteine, metallo and serine) have been directly or indirectly localized to the AIM of *H. contortus* [14], [15], [22]. Predicted homologs or orthologs of these *H. contortus* AIM peptidases are members of intestinal protein families (IntFam-241, [12]) found to be conserved among this parasite, *A. suum* and *C. elegans*. Nucleotide sequences for these *H. contortus* AIM proteins encode signal peptides, but not always hydrophobic sequences that are expected for integral membrane proteins, suggesting a peripheral association with the AIM. EST cluster sequences that encode the *A. suum* IntFam-241 homologues of *H.contortus* AIM peptidases were next mapped by BLASTN to *A. suum* gene models from the published *A. suum* genome sequences [18]. The *A. suum* gene and corresponding protein designations for these IntFam-241 peptidases are listed in Table 1. Accordingly, we hypothesized that the deduced *A. suum* proteins listed are AIM or IL peptidases.

**Table 1:** IntFam-241 proteins related to *Haemonchus contortus, Ascaris suum* and *Caenorhabditis elegans* intestinal peptidases.

Family^1^	Protein^2^	Cluster^3^
A1	GS_12574	AS00118.cl;
		AS00709.cl
	GS_15316	AS05229.cl
	GS_19445	AS02056.cl
	***GS_21229***	AS00866.cl
C01	GS_06461	AS00443.cl;
		AS05277.cl
M1	GS_04166	AS01463.cl
M20	GS_16898	AS01671.cl
S28	***GS_16223***	AS00974.cl

1 Peptidase family as defined in the MEROPS data base [27]. ^2^Protein designation based on BlastX of translated protein sequences [18] using cDNA cluster sequence data [12]. ^3^ *Ascaris suum* intestinal cDNA clusters described in [12]. Proteins highlighted in bold-italics were not detected in fractions analyzed in this paper.

### *A. suum* intestinal peptidase activity

**Figure 1:**
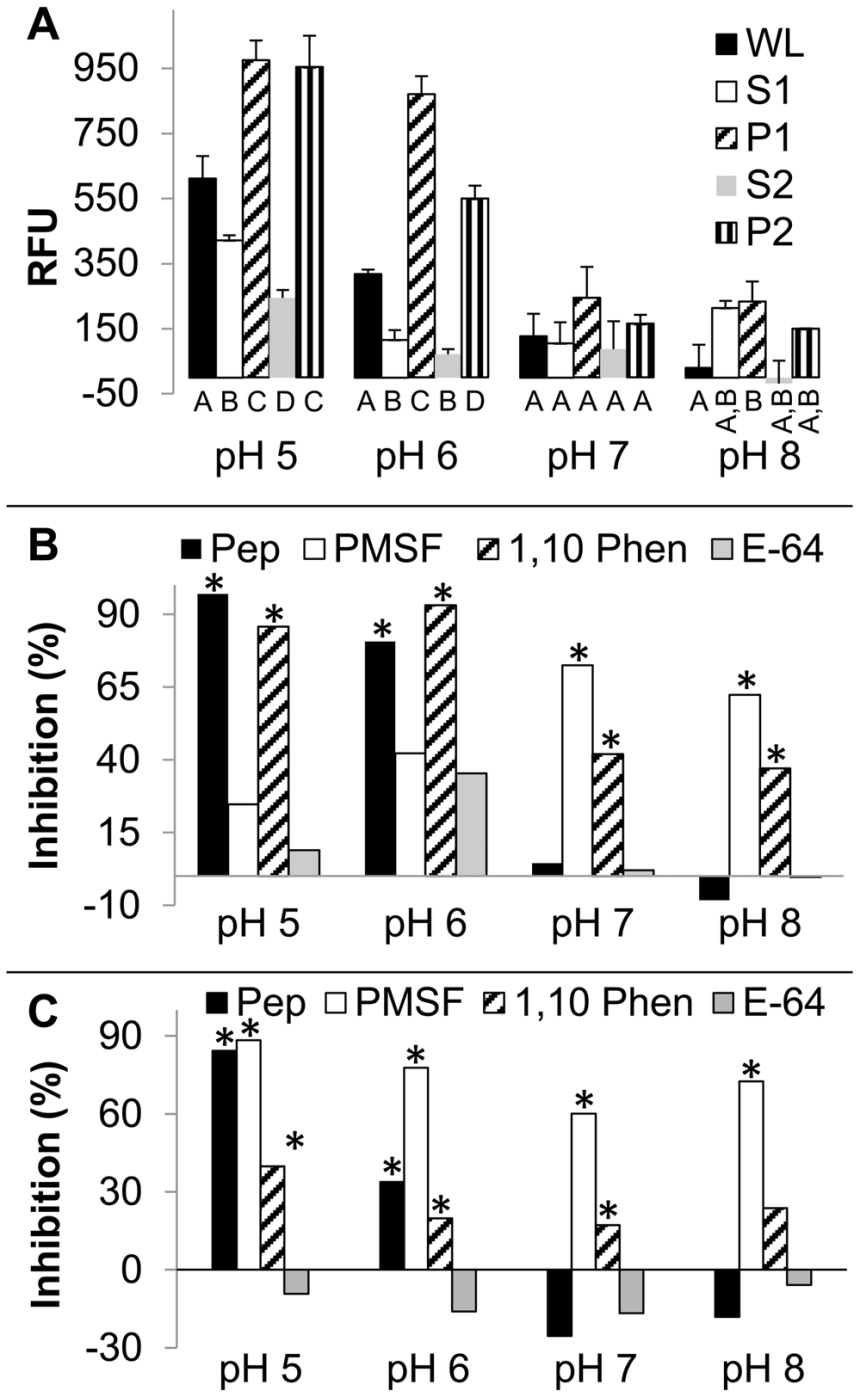
Intestinal peptidase activity. **A**. Whole intestinal cell fractionation of peptidase activity. Peptidase activity was determined using a Bodipy casein substrate with 4 µg of sample, as described in methods, for whole intestinal homogenates (WL), 5,000×*g* pellet (P1) and supernatant (S1), and a further 50,000×*g* pellet (P2) and supernatant (S2) derived from S1. All samples were solubilized in 1% TX-100 prior to running assays. pH at which reactions were run is indicated on the x axis. Peptidase activity (y axis) is reported as Relative Fluorescent Units (RFU) µg-protein^−1^. Means that differ from one another (p<0.05) are indicated by different letter designations (A, B, C, D) below the x axis, and were analyzed according to pH conditions. **B, C**. Inhibition of peptidase activity in the whole lysate (B) and P2 (C) fractions by pepstatin (aspartic proteases, Pep), phenylmethylsulfonyl chloride (serine peptidases, PMSF), 1,10 phenanthroline (metallopeptidases, 1, 10 Phen) and *trans*-epoxysuccinyl-L-leucylamido(4-guanidino)butane, L-*trans*-3-carboxyoxiran-2-carbonyl-L-leucylagmatine, N-(trans-epoxysuccinyl)-L-leucine 4-guanidinobutylamide (C1 cysteine peptidases (including cathepsin B) cysteine peptidases, E-64). Percentage inhibition (y axis) of the mean activity uninhibited compared to mean activity of the inhibited is shown for each pH (x axis) tested. An asterisk denotes treatments that were significantly different (p<0.05) from the untreated control group for each pH indicated. All assays were conducted with three replicates.

In previous research, intestinal homogenates and lysates from adult female *H. contortus* proved valuable for dissecting biological properties of AIM proteins, of which a large family of cathepsin B-like (CBL) cysteine peptidases comprise a prominent component [4], [13], [15], [22], [23]. This general approach was used to assess peptidase activity in the adult female *A. suum* intestine. Activity was detected in *A. suum* intestinal lysates using a Bodipy casein substrate predominately at pH 5.0 and 6.0, with lower activity at pH 7.0 and 8.0 (Fig. 1A).

The potential for *A. suum* peptidases to exist as membrane associated proteins was determined by enrichment for peptidase activity in pellet fractions P1 (large debris) and P2, (5,000 to 50000×*g*) (Fig. 1A). Although not exclusive to this fraction, the results show that intestinal peptidase activity was enriched in both P1 and P2 pellets relative to lysate of whole intestine or supernatant fractions in tested, which is likely to reflect membrane associated proteins.

General inhibitors effective against distinct peptidase classes were used to identify peptidases that might contribute to the activities detected in whole lysates (Fig. 1B) and the membrane enriched fraction P2 (Fig. 1 C). Inhibitors of aspartic, metallo and serine peptidases each caused inhibition of activity in intestinal lysates, and often in a pH dependent manner (Fig. 1B). For instance, the aspartic and metallo peptidase inhibitors, pepstatin and 1,10 phenanthroline, respectively, each inhibited activity at the more acidic range. In contrast, the serine peptidase inhibitor, phenylmethylsulphonylfluoride (PMSF) inhibited activity at neutral and basic pH conditions, while some inhibition by 1,10 phenanthroline was also observed under these pH conditions. No significant inhibition (p>0.05) was observed with an inhibitor of cathepsin B-like cysteine peptidases (E-64), and similar results were obtained with the general cysteine peptidase inhibitor, iodoacetamide (S1 Fig.). Results with the P2 pellet were similar to the lysate, except that PMSF showed greater inhibition under acidic conditions by comparison to the whole lysate.

The samples that were assayed are very complex. Although distinct effects were observed relative to pH and inhibitors, the results should be viewed as providing gross indications of peptidase activity in the intestinal homogenate and other fractions of interest, described below. The inhibitor results provided a sense of diversity relative to peptidase class and pH dependency for the peptidase activities detected, which will be evaluated in more detail by mass spectrometry in subsequent sections. Other intestinal fractions that will be described below were assayed for peptidase activity when incubated with Bodipy casein. However, use of denaturants with several samples described below introduced constraints on interpretations of those results. Nevertheless, inhibitors were routinely tested at pH 5.0 to assess peptidase involvement in producing signal in the assays.

### Concanavalin A binding proteins from *A. suum* intestine

**Figure 2:**
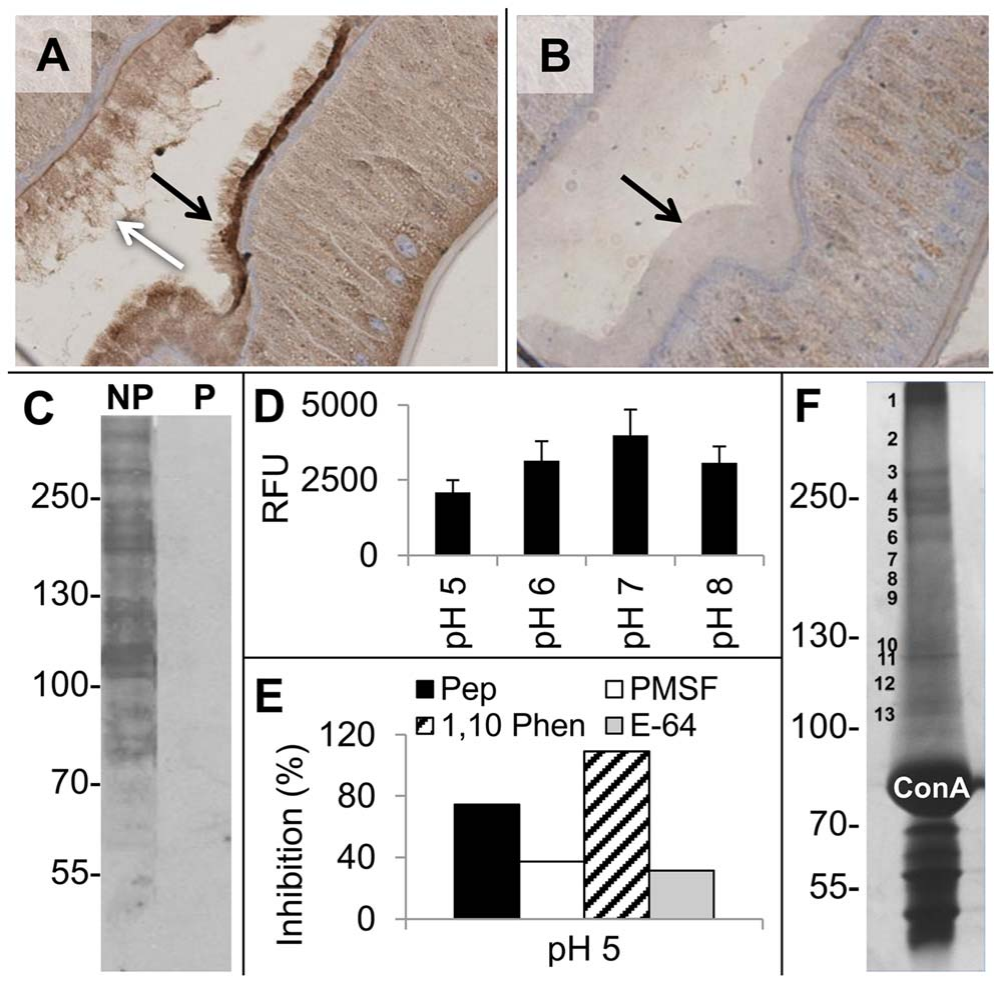
Concanavalin A (ConA) binding proteins from Adult *A. suum* intestine. **A, B**. Histological sections were treated without (A) or with sodium periodate (B), as described in Methods, and then incubated with the ConA-horse radish peroxidase conjugate (ConA-HRPO). Binding was localized by histochemical detection. Black arrows point to intestinal microvilli, the white arrow points to material extending from microvilli into the lumen. **C**. Intestinal proteins separated by non-reducing SDS-PAGE were transferred to nitrocellulose filters, and then untreated (NP) or treated (P) with sodium periodate. Filters were incubated with Con A-HRPO and ConA binding bands localized by chemiluminescence. Molecular weight markers are indicated on the left of the panel. **D**. Peptidase activity was evaluated for intestinal proteins bound to ConA-agarose beads, as described in Methods, and conducted at the pH conditions indicated on the x axis. Relative fluorescence units (RFU) µg-protein^−1^ from hydrolysis of Bodipy casein is indicated on the y axis. A 95% confidence interval was constructed for group means at each pH tested, which in each case was greater than zero. **E**. Inhibition of peptidase activity isolated on ConA agarose beads. Assays were conducted at pH 5.0. Percentage inhibition indicated on the y axis was determined with inhibitors, as described in Figure 1, and means for each inhibitor treatment tested were significantly lower (p<0.05) than the uninhibited control group. All peptidase assays were conducted with three replicates. **F**. Coomassie blue stained SDS PAGE gel, as in panel C, of intestinal proteins isolated on ConA agarose beads. ConA, indicates heavy ConA proteins released from the beads. No bands were excised for mass spectrometry at or below this position in the gel.

Several known proteins, including peptidases, located on the *H. contortus* AIM are glycosylated [4], [14]. Hence, we investigated the use of a lectin, concanavalin A (ConA), to identify glycosylated proteins on the *A. suum* AIM. Glycans located on the *A. suum* AIM were previously detected by ConA [24], and this observation was confirmed here (Fig. 2A, B). Pretreatment of tissue with periodate prevented binding of ConA to the AIM surface, indicating specificity of ConA binding to the *A. suum* glycans. ConA binding was most obvious on and at the base of the *A. suum* AIM. There was also evidence of ConA binding to material that appeared loosely attached to the AIM and extended into the lumen, which may reflect insoluble cellular material on the AIM surface that is released from the AIM into the lumen.

ConA was used to probe blots of SDS-PAGE gels in which proteins of intestinal lysates were separated (Fig. 2C). A large number of protein bands were identified that ranged in M*r* from 14 to over 225 kDa. Binding of ConA to these bands was inhibited by pretreatment of the blot with periodate, which supported the dependence of binding on the presence of periodate-sensitive glycans.

Next, ConA-agarose beads were used to isolate ConA binding proteins from Triton X-100 lysates of the intestine. Proteins that remained on the beads following washes hydrolyzed Bodipy casein at pHs that ranged from 5.0 to 8.0 (Fig. 2 D). In this case, inhibitors of aspartic, cysteine, metallo and serine peptidases significantly reduced peptidase activity associated with beads. (Fig. 2 E). Although the extended time required for ConA binding may have differentially affected the various peptidase activities, the results support that peptidases were isolated on ConA beads. On the contrary, methods used to isolate ConA binding proteins for analysis by mass spectrometry included SDS treatment, and no peptidase activity was detected with beads from these preparations.

Collectively, the results showed that i) ConA bound both to the AIM and IL content of *A. suum,* ii) multiple ConA binding proteins exist in the *A. suum* intestine, and iii) intestinal peptidase activity was isolated on ConA-agarose beads.

### Peptidase activity in perfusates of the *A. suum* intestinal lumen

**Figure 3:**
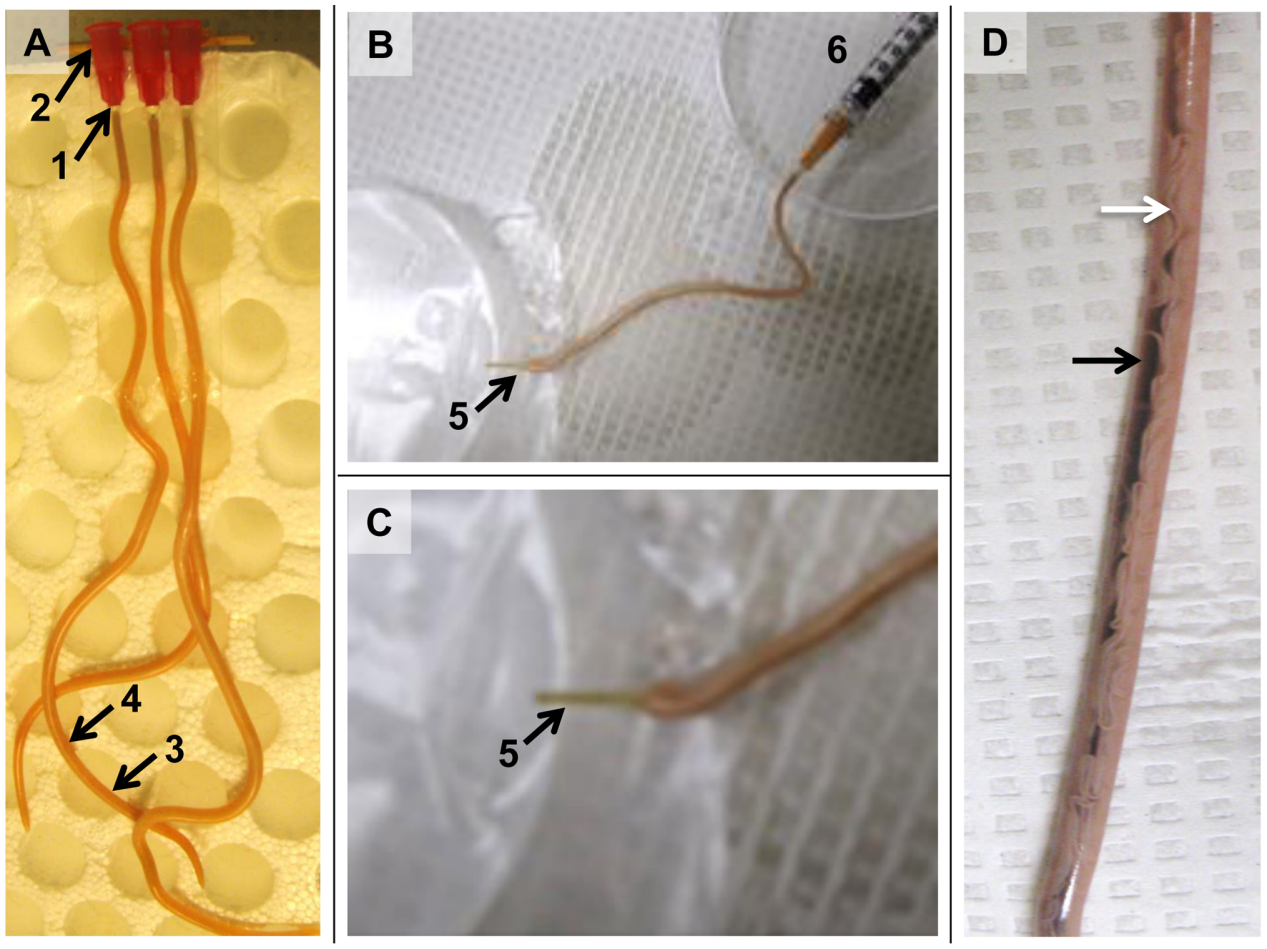
Cannulation of the Ascaris suum intestine. **A**. Three cannulated female *A. suum* with intact posterior ends. Numbers and arrows refer to steps in the cannulation process: 1, removal of the anterior end below the esophagus with a scalpel; 2, insertion of the blunt needle cannula (25 g), with superglue gel applied to the cannula, into the intestinal lumen; 3, removal of the posterior 1/6^th^ of the worm; 4, resection of the body wall to expose the intestine, as in panels B and C. **B**. Cannulated worm processed as in A, but with resected posterior body and exposed posterior region of the intestine (arrow, 5), laid onto parafilm for collection of perfusate. A syringe (6) is attached to the cannula hub to deliver perfusate. **C**. Enlargement of the exposed posterior region of the intestine is shown in panel B. **D**. Cannulated worm, with intact posterior end, injected with methylene blue dye. White arrow points to reproductive organs within the pseudocoelomic body cavity, black arrow points to the intestine filled with dye. Note that the dye is confined to the intestine.

The foregoing experiments provided general information on characteristics of *A. suum* intestinal proteins that may include AIM or IL glycoproteins and peptidases. Cannulation and perfusion of the intestinal lumen provided a more direct approach uniquely supported by the large size of *A. suum*, as compared to other nematodes such as *H. contortus* and *C. elegans*, Fig. 3 A-C indicates the steps utilized in cannulation to perfuse the *A. suum* intestine. Injection of dye into the intestinal lumen of worms with an intact posterior end demonstrated confinement of dye to the lumen (Fig. 3D).

IL content was first collected by perfusion with phosphate buffered saline (PBS perfusate). This perfusion was followed in the same worms by perfusion with PBS containing 4 M urea (4MU perfusate). The urea chaotrope was perfused because ConA binding was most evident on the AIM, by comparison to the IL, suggesting that at least some predicted AIM glycoproteins (peptidases) are peripheral AIM proteins that may be solubilized by 4MU and collected for analysis in this perfusate.

**Figure 4:**
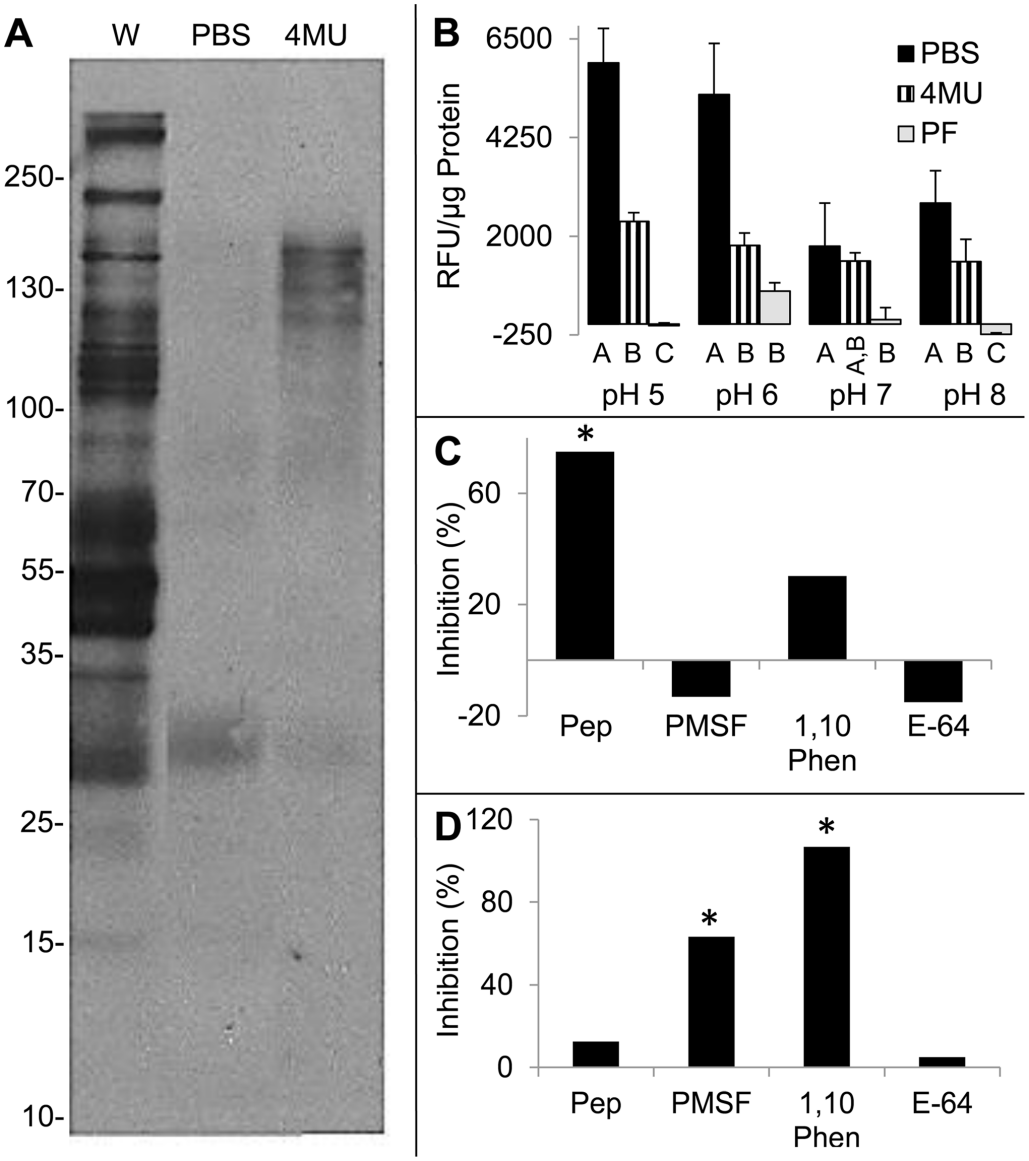
Peptidase activity in intestinal perfusates. A. ConA-HRPO blot of proteins obtained from whole intestinal lysate (W), and PBS (P) or 4 M urea (4MU) perfusates of the intestinal lumen after separation by non-reducing SDS-PAGE and transfer to nitrocellulose. **B**. Samples (4 µg) of the PBS and 4MU perfusates, and pseudocoelomic fluid (Pf) were incubated with bodipy casein in peptidase assays, as described in Figs. 1 and 2. Measurements in Relative fluorescence units (RFU) µg-protein^−1^ are shown on the y axis. Means that differ from one another (p<0.05) are indicated by different letter designations (A, B, C) below the x axis, **C, D**. Inhibition of peptidase activity in PBS (B) and 4MU (C) perfusates. Assays were conducted at pH 5.0. Percentage inhibition indicated on the y axis was determined for with inhibitors, as described in Fig. 1. Means for each inhibitor treatment that were significantly lower (p<0.05) than the uninhibited control group are indicated by an asterisk. All peptidase assays were conducted with three replicates.

Blots of isolated proteins in both perfusates were probed with ConA (Fig. 4A), which showed somewhat similar banding patterns overall between the PBS and 4MU perfusates, but with different relative abundances of ConA binding proteins in each. Periodate pre-treatment of the nitrocellulose filter (blot) eliminated detectable ConA binding to protein bands from both perfusates.

In peptidase assays, mean activity of the PBS perfusate was generally higher than the 4MU perfusate and the pseudocoelomic fluid (PF), in which negligible peptidase activity was detected, except for the low level at pH 6.0. The activity in the PBS perfusate at pH 5.0 was largely inhibited by pepstatin (Fig. 3B, C). Despite treatment with denaturant, peptidase activity was detected in the 4MU perfusate under all pH conditions tested. Mean fluorescence was significantly higher than PF at both pH 5.0 and 8.0. In contrast to the PBS perfusate, significant inhibition was observed with PMSF and 1,10 phenanthroline at pH 5.0 (Fig. 3F). Although these results suggest enrichment for different classes of peptidases, between the PBS and 4MU perfusates, inhibitory effects of urea on peptidases in the 4MU fraction also seems likely, rendering this point inconclusive.

These results showed that 1) PBS and 4MU intestinal perfusates contained ConA binding proteins, 2) ConA binding proteins were differentially obtained from the intestine according to specific perfusion conditions, and 3) both perfusates contained peptidase activity that was distinct from potential contamination by PF.

### Mass spectrometric analysis of *A. suum* intestinal fractions

Proteins from each of the fractions described above (ConA agarose beads, PBS and 4MU perfusates, and P2 pellet) were analyzed by LC-MS/MS mass spectrometry. A complete listing of results is provided in S1 Table. The smallest subset of proteins identified was generated with proteins eluted from ConA agarose beads (full set in S2 Table, peptidases in Table 2). These bead-isolated proteins were separated by PAGE, and ConA binding bands located on nitrocellulose filters were then excised from corresponding gels for analysis (as described in the methods). Twenty seven proteins were identified, most individual proteins of which were confined to one or two unique PAGE gel slices (S2 Table). Eighteen of the 27 proteins fell into the two major functional categories of peptidases (10) and O-glycosyl hydrolases (eight), of which the glycosyl hydrolases may be related to saccharidase activity previously detected in *A. suum* intestinal brush border preparations [25], [26]. Only three proteins (GS_16354, GS_12574, GS_21785) from the entire ConA set were not detected in either of the perfusate fractions, and only one (GS_21785) was absent from both the perfusate and P2 pellet fractions (Table 2). For proteins detected in both the ConA binding proteins and the perfusates, representation was often the highest in the 4MU versus PBS perfusate, which might be expected for peripheral membrane proteins. Therefore, the ConA isolated proteins identified appear to account, at least in part, for ConA binding proteins located *in situ* on the AIM, and for peptidase activity associated with ConA agarose beads and perfusates.

**Table 2:** All predicted *Ascaris suum* peptidases in intestinal fractions and identified by mass spectrometry.

Peptidase^1^	Family^2^	Fraction^3^					Compartment^4^				Expression^6^	
		ConA	PBS	4MU	P2	Pf	SP	NC	TM	Secreted^5^	Female	Male
GS_23920	-	Y	2	2	5	0	-	Y	-	-	1	1
GS_12574	A01A	Y	0	0	8	0	Y	-	-	Y	1	1
GS_14901	A01A	Y	1	5	7	0	Y	-	-	-	1	1
GS_15316	A01A	Y	3	2	8	0	Y	-	-	-	1	1
GS_19445	A01A	Y	1	4	7	0	Y	-	-	-	0	1
GS_04166	M01	Y	6	9	36	0	-	Y	-	Y	1	1
GS_05584	M01	Y	0	6	18	0	-	Y	-	Y	1	1
GS_05746	M01	Y	8	9	47	0	Y	-	-	Y	1	1
GS_16285	M01	Y	1	2	36	0	-	Y	-	Y	1	1
GS_08219	M13	Y	11	212	64	1	Y	-	-	Y	1	1
GS_03841	S10	Y	3	4	55	0	Y	-	-	Y	1	1
GS_22704	S10	Y	0	6	44	0	-	Y	-	Y	1	1
GS_23459	-	0	3	1	13	0	-	Y	-	Y	1	1
GS_00518	M01	0	1	0	4	0	-	-	-	-	1	1
GS_03428	A01A	0	2	6	0	0	Y	-	-	-	0	0
GS_06461	C01A	0	1	0	0	0	-	Y	-	-	1	1
GS_02555	M01	0	2	8	27	0	-	Y	-	Y	1	1
GS_21518	M01	0	2	2	9	0	-	Y	-	-	1	1
GS_13515	M01	0	1	9	32	0	-	Y	-	Y	1	1
GS_19140	M13	0	5	43	41	0	-	Y	-	Y	1	1
GS_10348	M13	0	3	12	55	0	-	Y	-	-	1	1
GS_02701	M13	0	1	1	9	0	-	-	Y	Y	1	-1
GS_16898	M20A	0	2	6	9	0	-	-	-	Y	1	1
GS_11178	S28	0	1	1	9	0	Y	-	-	-	1	1
GS_09307	M01	0	0	4	25	0	-	Y	-	Y	1	1
GS_14497	M17	0	0	2	0	0	Y	-	-	-	0	1
GS_16008	M20A	0	0	3	22	0	-	Y	-	-	1	1
GS_09676	S10	0	0	1	3	0	-	-	-	-	1	1
GS_05016	T01A	0	0	1	0	0	-	Y	-	-	-1	0
GS_11477	-	0	0	0	3	0	-	-	-	-	1	1
Liv_01603	S09	0	0	0	1	0	-	Y	-	-	1	0
GS_01798	C13	0	0	0	2	0	Y	-	-	-	1	1
GS_03481	C19	0	0	0	1	0	-	Y	-	-	0	0
GS_11940	C95	0	0	0	4	0	Y	-	-	-	1	1
GS_18334	C95	0	0	0	3	0	-	Y	-	-	1	1
GS_13060	M67A	0	0	0	1	0	-	Y	-	-	-1	0
GS_16309	S09	0	0	0	3	0	-	Y	-	-	1	1
GS_01230	S09	0	0	0	2	0	Y	-	-	-	1	1
GS_19153	S09	0	0	0	2	0	-	-	Y	-	0	1
GS_01127	S10	0	0	0	2	0	-	Y	-	-	0	0
GS_06945	S10	0	0	0	1	0	Y	-	-	-	1	1
GS_14352	S10	0	0	0	1	1	Y	-	-	-	-1	-1
GS_03565	S12	0	0	0	2	0	Y	-	-	-	1	1
GS_00889	S16	0	0	0	1	0	-	-	Y	-	0	0
GS_08933	S26B	0	0	0	1	0	-	Y	Y	-	0	0
GS_04641	S28	0	0	0	3	0	Y	-	-	-	1	1
GS_07735	S28	0	0	0	3	0	Y	-	-	-	1	1
GS_16223	S28	0	0	0	3	0	-	Y	-	-	1	1
GS_23795	S28	0	0	0	1	0	Y	-	-	-	1	1
GS_11889	S33	0	0	0	6	0	-	-	Y	-	-1	-1

1 Protein designation from translated protein sequences [18], except Liv_01603 identified in an L4 gene library. ^2^Peptidase family as defined in the MEROPS data base [27]; -, unassigned family, see more complete annotation in Table S1. ^3^Fractions in which proteins were identified (numbers refer to quantity of mass spectra): ConA, Concanavalin A (Y, yes, number of spectra are listed in Table S2); PBS or 4MU, PBS or 4 M urea intestinal perfusates, respectively; P2, 5K-50K x g intestinal pellet; Pf, pseudocoelomic fluid. ^4^SP, signal peptide; NC, Non-classical secretion; TM, transmembrane. ^5^designated as a component of the secretome [29]. ^6^Transcript abundance that is: 1, relatively more abundant in the intestine; 0, not detectably different in the intestine; and -1 less abundant in the intestine compared to other tissues investigated [30].

A more complex group of peptidases was detected in perfusate samples (Table 2). These samples were also compared to LC-MS/MS results obtained for the PF, a most likely contaminant of the perfusate samples. Predicted peptidases identified in the PBS and 4MU perfusates by LC-MS/MS showed both similarities and differences. Perfusate proteins annotated as peptidases corresponded to aspartic, cysteine, metallo, serine carboxy and threonine peptidases, or unassigned peptidases, according to MEROPS [27]. These proteins were represented in one or both perfusates, although representation of mass spectra was better in the 4MU perfusate for many proteins shared with the PBS perfusate. In total, 29 peptidases were detected in ConA and perfusate fractions (Table 2). All but four of these were also detected in the P2 pellet. The largest group of peptidases identified in the Con A and perfusate fractions were categorized as metallopeptidases (M01, nine; M13, four; M17, one; M20, two), followed by aspartic peptidases (A01, five), serine carboxy peptidases (S10, three; S28, one), cathepsin B-like cysteine peptidases (C01, one), threonine peptidase (T01A, one) and unassigned amino peptidase N (-, two).

Peptidase classes represented in the perfusates and ConA binding fraction were largely consistent with our previously published predictions (Table 1), although the results significantly expanded the subclasses, increased the number and provided direct evidence for *A. suum* AIM and IL peptidases identified relative to these predictions (Table 3). The relatively high representation of some peptidase sequences in the 4MU fraction and occurrence in the ConA fraction is consistent with localization to the AIM as peripheral membrane proteins.

**Table 3:** Summary of predicted *Ascaris suum* peptidases identified in Concanavalin A (ConA) binding and intestinal perfusate fractions.

Family	ConA^1^	Perfusates^2^
A1	***GS_12574^3^***	GS_03428
	GS_14901	
	***GS_15316***	
	***GS_19445***	
C01		***GS_06461***
M01	***GS_04166***	GS_02555
	GS_05584	GS_21518
	GS_05746	GS_13515
	GS_16285	GS_09307
		GS_00518
M13	GS_08219	GS_19140
		GS_10348
		GS_02701
M17		GS_14497
M20		***GS_16898***
		GS_16008
S10	GS_03841	GS_09676
	GS_22704	
S28		GS_11178
T01		GS_05016
Unassigned	GS-23920	GS_23459

1 Proteins listed as ConA and detected in Perfusates are listed in the ConA column. ^2^Proteins unique to Perfusates are listed in the Perfusates column. ^3^A. suum proteins predicted as intestinal peptidases in Table 1 are highlighted in bold-italics

Although information from the P2 fraction was not specific regarding compartmentalization, this fraction contained 21 additional putative peptidases, including seven S10 and S28 serine carboxypeptidases, which may also function on the AIM or in the IL.

In contrast to *H. contortus* in which cathepsin B-like cysteine peptidases make up a prominent fraction of AIM-IL peptidases [15], [23], cysteine protease activity was variably indicated in inhibitor assays and a single peptide was detected in the PBS perfusate for an *A. suum* cathepsin B-like (CBL) cysteine peptidase. Thus, *A. suum* CBLs appear to represent a relatively minor constituent of *A. suum* AIM and IL digestive peptidases.

Also, based on acidic preference of serine carboxypeptidases, no AIM or IL peptidases were identified that would obviously account for the apparent serine peptidase activity detected at pH 8.0. Although host trypsin and chymotrypsin apparently localize to the *A. suum* intestine [28], use of porcine trypsin for the mass spectrometry analysis obviated clarification based on detection of this protein. Nevertheless, peptides of porcine chymotrypsin were also detected in perfusates, raising the possibility of host serine peptidase activity in perfusates analyzed at higher pHs.

Several of the peptidase protein sequences were predicted to have signal peptides for secretion, or are predicted non-classical secretory proteins, each of which is consistent with compartmentalization in the IL or on the AIM. However, neither of these two characteristics was predicted for three of the ConA and perfusate peptidases based on existing *A. suum* protein models (Table 2). A recent publication on the *A. suum* secretome [29] identified 10 of the ConA and perfusate peptidases identified here as excretory-secretory products of adult female *A. suum* (Table 2), six of which lack apparent signal peptides, and five of those were classified as non-classical secretory proteins. While our results clarify the likely origin of these “secretory” products, their localization in excretory-secretory products is also consistent with secretion from the AIM. It is possible that inaccurate gene models account for lack of detectable signal peptides on some proteins and for some discrepancies between bioinformatics annotation and experimental results.

We also detected intestinal transcripts for each of the predicted peptidase genes under evaluation [30] and many of these showed relative abundances that are equal to or greater in intestinal cells compared to other tissues investigated (Table 2).

### *A. suum* peptidases accounted for by peptidases in intestinal fractions

We next assessed representation of all *A. suum* peptidases that can be accounted for by intestinal peptidases detected in the compartments under investigation. All peptidases annotated in the entire proteome were evaluated relative to transcript expression and over-expression of transcripts in the intestine compared to other adult tissues, based on previous measures [30], and then, those peptidases detected by LC-MS/MS in fractions analyzed here. The total number of peptidases for which intestinal transcripts have been detected represent from 78 to 100% of all peptidases annotated in the *A. suum* proteome (Table 4). However, the range of percentages decreased substantially (5 to 26% of all peptidases) when considering peptidase for which transcripts were relatively overexpressed in intestinal tissues. In addition, representation of peptidases identified by LC-MS/MS was much higher for the “overexpressed” group. For instance, the number of peptidases detected in both perfusates account for a high percentage of metallo (68%), aspartic (100%) and unassigned (100%) peptidases classified in the “overexpressed” group. Whereas serine and cysteine peptidases classified as “overexpressed” were less well represented in the perfusates (6 and 13%, respectfully), representation of serine peptidases improved when data for the P2 pellet fraction was included (62%).

**Table 4:** Peptidase intestinal gene expression and presence among *A. suum* proteomic datasets.

Peptidase Class^1^	Total number in genome	Number expressed in intestine^2^	Number over- expressed in intestine^2^	Supported by proteomic detection^3^ (also overexpressed in the intestine)				
				ConA	PBS	PBS+4MU	P2	Total
Metallo	148	116	22	5 (5)	11 (11)	15 (15)	15 (14)	16 (15)
Serine	120	105	32	2 (2)	3 (2)	5 (4)	18 (13)	20 (13)
Cysteine	91	85	16	0	1 (1)	0	4 (3)	6 (4)
Threonine	19	19	2	0	0	1	0	1
Aspartic	19	13	4	4 (4)	4 (3)	4 (3)	4 (4)	5 (4)
Unknown	21	18	1	0	1	0	0	1
Any	418	356	77	11 (11)	20 (17)	25 (21)	41 (34)	49 (36)

1 Protease classes identified using MEROPS web server [27]; ^2^Intestinal Expression and overexpression based on previous study [30]; ^3^Data collected from this study.

The results show that peptidases in perfusates accounted for a high percentage of intestinal peptidases identified in the group with transcripts comparatively overexpressed in the *A. suum* intestine. While but a relatively small fraction of all prospective intestinal peptidases, this connection between AIM-IL peptidases and the “overexpressed” group conveys relative importance of these proteins for the intestine and the parasite.

### Other *A. suum* intestinal perfusate proteins identified by mass spectrometry

Although peptidases were used as markers for the IL and AIM compartments, fractions containing these proteins were expected to include IL and AIM proteins that have other functions, which is evident in S1 Table. More detailed analysis will be reported on these other proteins a separate publication.

## Discussion

Parasitic nematodes present numerous challenges for conducting research on individual worm tissues, and even more so on individual cells. Such is the case for biological questions pertaining to the intestine and intestinal cells of these pathogens. Here we describe progress towards comprehensive identification of proteins that function in the IL and/or on the AIM of adult female *A. suum*, which is one of a relatively few parasitic nematodes with sufficient size to support the progress reported by using relatively straightforward perfusion methods. Knowledge of *H. contortus* AIM peptidases gained from past research guided efforts to identify *A. suum* homologs (likely orthologs or paralogs) that were detected in ConA binding fractions and intestinal perfusates. Collective observations reported here indicated that most of the predicted aspartic, metallo and serine carboxy peptidases identified in Table 1, are indeed *A. suum* glycoproteins that function in the IL and/or on the AIM. Moreover, testing of this hypothesis led to identification of a greatly expanded set of apparent peptidases that function in these compartments, while also identifying many proteins with other functions that are likely sited in these compartments.

The foregoing conclusions are supported by multiple considerations, different subsets of which apply to different peptidases; i) detection of peptidase activity in the PBS and 4MU perfusates of the *A. suum* IL, ii) detection by LC-MS/MS of proteins in the perfusates that are predicted to have relevant peptidase properties, iii) localization of these peptidases, or subsets of peptidases in fractions enriched for membranes (P2 pellet) and glycosylated proteins (ConA binding proteins), iv) evidence of classical or non-classical secretion for proteins encoded by corresponding *A. suum* genes of many, but not all of the these proteins [30].

Many peptidases were detected in multiple fractions and those preferentially detected in the 4MU perfusate are also candidate peripheral AIM proteins. Their detection in the lumen could reflect an origin as a peripheral membrane protein on the AIM with subsequent release into the lumen as part of a normal process of protein turnover. A peripheral association is supported by other characteristics including detection in the ConA and/or P2 fractions, presence of a signal peptide and lack of a transmembrane domain. While none of the individual characteristics is sufficient alone, in combination these characteristics increase the probability for a given protein to have a peripheral membrane association. Nevertheless, apparent lack of a signal peptide must be tempered in these considerations pending improvement of *A. suum* gene models. We expect that signal peptides will be recognized for additional proteins identified here with future refinement of these gene models.

AIM and IL peptidases have proven valuable as targets in control strategies against parasitic nematodes of humans, animals and plants [26], [31]. Approaches have included i) vaccination, and ii) delivery of peptide inhibitors and double stranded RNA to disrupt intestinal peptidase functions in multiple parasitic nematodes [31]-[33]. Results presented here provide a broad and deep assessment of peptidases that *A. suum* likely relies upon for digestion of ingested proteins. The number and diversity of peptidases identified was not necessarily expected for a parasite that consumes intestinal content already digested to a large extent by the host. The results indicated that the intestine of *A. suum* is rich in peptidases actively engaged in digestion of proteins obtained from the host intestinal content.

Comparisons made at the whole genome and intestinal transcriptome levels provided quantitative context for the subset of *A. suum* peptidases that are likely involved in protein digestion within the adult female intestinal lumen. This subset reflects a relative small fraction of the entire set of prospective peptidases derived from *A. suum* genome and intestinal transcriptome analyses [18], [30]. Many of the peptidases identified in perfusates are encoded by transcripts determined to be relatively overexpressed in intestinal cells as compared to other tissues [30]. This combination of data adds confidence that these proteins contribute significant roles in the *A. suum* intestine and indicates that the up-regulation of expression of these peptidases is an important aspect of development in intestinal cells.

The foregoing knowledge will contribute significantly to research directed at disruption of protein digestion in *A. suum* by chemical or immunological means. For instance, in contrast to some other plant and animal parasitic nematodes [23], [32], the single cathepsin B-like cysteine peptidase detected had low representation in intestinal samples analyzed and therefore may be a low value target for disrupting protein digestion in *A. suum*. Alternatively, aspartic peptidases represent a major group of *A. suum* endopeptidases implicated in protein digestion, although the moderately large number identified will be an important consideration relative to inhibitory methods that rely on antibodies or RNA interference. Several of the aspartic peptidases were annotated with similarity to necepsins, which are vaccine candidates for hookworms [7]. Likewise, a group of 16 apparent IL-AIM metallopeptidases were identified in intestinal perfusates, and far more than is known from any other nematode species. Predicted amino (M01) and endo (M13) peptidases were prominent components of this class, and mass spectra for two apparent M13 peptidases were overrepresented in perfusates (GS_08219 and GS_19140), perhaps reflecting the relative biological importance of these peptidases. Serine carboxypeptidases present a less complex picture, although mass spectra specific to the P2 fraction identified additional serine carboxypeptidases, which may foretell a more complex picture. While the value of these proteins as vaccine targets is unclear, transcripts encoding serine carboxypeptidases are highly abundant in *H. contortus* intestine [22], [23] and these peptidases have been implicated as vaccine candidates against this parasite [34]. Accordingly, the ability to perfuse the *A. suum* intestine offers an approach for testing methods of inhibition *in vitro* using perfusates isolated from the worm and *in vivo* by perfusing inhibitors into the intestine contained within otherwise intact worms.

The similarities in profiles of IL-AIM peptidases among diverse species offer potentially broad application of results, while differences represent adaptations that may be explained in several ways: 1) influence of trophic niche (food from host intestinal content versus blood) between species like *A. suum* and *H. contortus*, respectively; 2) unique evolutionary solutions for nutrient acquisition among phylogenetic lineages; or 3) combinations of these factors. For instance, our results confirm previous findings [23] that *A. suum* apparently expresses a single intestinal CBL, which has a relative minor role in protein digestion, whereas *H. contortus* expresses numerous intestinal CBLs that appear to have a prominent role in nutrient digestion. Given the high number of IL-AIM metallopeptidases, *A. suum* may have comparatively high representation of this class of peptidase among nematodes, although more comparative information is needed to address this point. While differences may clarify biological distinctions among individual pathogens, it will also be of interest to learn at what phylogenetic level differences of this kind have been acquired. Because adaptations related to nutrient acquisition likely represent major determinants in parasite evolution, additional information here may clarify factors critical for the evolution of parasite lineages and/or species.

In addition to AIM and IL peptidases, our approach has provided a major step towards comprehensive identification of IL proteins free in the lumen because most (all) of these proteins are expected to be collected in the PBS perfusate. Use of 4 M urea was expected to extract many peripheral AIM proteins, but not integral membrane proteins, which are likely to have gone largely unidentified in this analysis. Nevertheless, the ability to perfuse the intestine should facilitate use of other methods to further elucidate *A. suum* AIM proteins. The ability to perfuse the intestine has also expanded experimental capabilities to directly investigate *in vivo* intestinal functions that serve the IL, AIM and worm in general. Capability of this kind is lacking for most other nematodes, including *C. elegans*, and represents an important adjunct to experimental dissection of intestinal cell functions of relevance to all nematodes.

Beyond peptidases, proteins predicted to perform numerous other functions were identified in the PBS and 4MU perfusates. While analysis of these proteins is presented in more detail elsewhere, some may have roles that are complementary with the peptidases discussed here. An example is the VHA subunits detected (A, B, C, D, E and G) in the 4MU perfusate which may function to create acidic conditions in the *A. suum* intestinal lumen and aid in digestion of ingested nutrients. VHAs translocate H+ across membranes and have been implicated as AIM determinants of acidic pH in the intestinal lumen of *C. elegans* [35]. The peptidase activity detected at more alkaline pH notwithstanding, an acidic pH supported peptidase activities from intestinal fractions inclusive of apparent aspartic, serine and metallo peptidases, which is consistent with *A. suum* proteins identified in intestinal perfusates. Similarly, *A. suum* intestinal saccharidases associated with AIM brush border preparations (microvilli) had acidic pH preference [25], [26]. This association with the brush border was suggested to reflect compartmentalization of carbohydrate digestion at the AIM surface that facilitates localized coupling of nutrient transport across the AIM [26]. Our results extend this possibility to include coupling of protein digestion and peptide transport that is facilitated by close association on the AIM. Although acidic conditions may prevail throughout the *A. suum* intestinal lumen, detection of intestinal peptidase activity at neutral and basic pHs prevents exclusion of possible regional variation of pH along the length of the lumen. In any case, this research provides new capabilities to develop and investigate questions on mechanisms of nutrient acquisition by *A. suum*, and by extension, methods to inhibit those processes.

Research leading to the progress reported here stemmed from comparative analysis of intestinal cDNAs from *A. suum*, *H. contortus* and *C. elegans*. The large size of *A. suum* uniquely facilitated identification of many proteins that appear to be sited in the intestinal lumen and on the AIM. How the current findings apply to other parasitic nematodes is now of interest to determine. The perfusion method described here cannot be effectively applied to address this question in many other species of nematodes important to the health of humans and agricultural species. Nevertheless, transcriptomic/genomic methods provide a means to link experimental results obtained with *A. suum* to other nematodes of medical importance. Accordingly, computational research is ongoing to elucidate orthologous intestinal functions of basic importance among nematodes ([12] and unpublished research), which will complement well the progress that can be made using the experimental intestinal model that is provided by *A. suum*.

## Supporting Information

S1 Fig Incubation of the *A.suum* whole intestinal lysate (closed bar) and P2 (open bar) fractions with iodoacetamide (100 µM). Peptidase assays were completed exactly as described in Fig. 1.
(DOCX)

S1 Table *Ascaris suum* protein annotation and proteomics data.
(XLSX)

S2 Table *Ascaris suum* intestinal proteins isolated on Concanavalin A agarose beads and identified by mass spectrometry.
(DOCX)

S1 Text Detailed materials, methods and analysis.
(PDF)
