# Supplementary material for: Peptidases Compartmentalized to the Ascaris suum Intestinal Lumen and Apical Intestinal Membrane
Source: PLoS Negl Trop Dis. 2015 Jan 8;9(1):e3375. doi: 10.1371/journal.pntd.0003375 (PMC4287503; doi:10.1371/journal.pntd.0003375)
Supplement: S2 Table — Ascaris suum intestinal proteins isolated on Concanavalin A agarose beads and identified by mass spectrometry. (DOCX) [file pntd.0003375.s003.docx]

| **Supporting Table S2.** *Ascaris suum* intestinal proteins isolated on Concanavalin A agarose beads and identified by mass spectrometry. | | | | | | | | | | | | | | | | | | | | | | | |
| --- | --- | --- | --- | --- | --- | --- | --- | --- | --- | --- | --- | --- | --- | --- | --- | --- | --- | --- | --- | --- | --- | --- | --- |
|  |  | Other Fraction^3^ | | | |  | Compartment^4^ | | |  | M*r* (kDa)^5^ | | | | | | | | | | | | |
| Protein^1^ | Peptidase/  Glycosylase^2^ | PBS | 4MU | P2 | Pf |  | SP | NC | TM |  | 258 | 215 | 166 | 132 | 117 | 107 | 96 | 85 | 73 | 58 | 52 | 40 | 34 |
|  |  |  |  |  |  |  |  |  |  |  |  |  |  |  |  |  |  |  |  |  |  |  |  |
| GS_13055 | - | 0 | 13 | 72 | 0 |  | Y | Y | - |  | 10 | - | - | - | 8 | - | 7 | - | 6 | 9 | 8 | 7 | - |
| GS_14784 | - | 0 | 2 | 12 | 0 |  | - | Y | - |  | 3 | - | - | - | 1 | - | - | - | - | - | - | - | - |
| GS_15893 | G | 1 | 3 | 6 | 0 |  | - | Y | Y |  | 11 | - | - | - | - | - | - | - | - | - | - | - | - |
| GS_16354 | G | 0 | 0 | 6 | 0 |  | - | Y | - |  | 14 | - | - | - | - | - | - | - | - | - | - | - | - |
| GS_18934 | G | 2 | 2 | 7 | 0 |  | - | - | Y |  | 8 | - | - | - | - | - | - | - | - | - | - | - | - |
| GS_23879 | G | 12 | 27 | 58 | 0 |  | - | Y | - |  | 22 | - | - | - | - | - | - | - | - | - | - | - | - |
| GS_05584 | M01 | 0 | 6 | 18 | 0 |  | - | Y | - |  | - | - | 3 | 7 | 9 | - | - | - | 5 | - | - | - | - |
| GS_08219 | M13 | 11 | 212 | 64 | 1 |  | Y | - | - |  | - | - | 40 | - | - | - | - | - | - | - | - | - | - |
| GS_22704 | S10 | 0 | 6 | 44 | 0 |  | - | Y | - |  | - | - | 9 | - | - | - | - | - | - | - | - | - | - |
| GS_16285 | M01 | 1 | 2 | 36 | 0 |  | - | Y | - |  | - | - | - | 2 | - | - | - | - | - | - | - | - | - |
| GS_03841 | S10 | 3 | 4 | 55 | 0 |  | Y | - | - |  | - | - | - | - | 27 | - | - | - | - | - | - | - | - |
| GS_04166 | M01 | 6 | 9 | 36 | 0 |  | - | Y | - |  | - | - | - | - | 24 | - | - | - | - | - | - | - | - |
| GS_10842 | - | 0 | 2 | 6 | 0 |  | - | - | - |  | - | - | - | - | - | 9 | - | - | - | - | - | - | - |
| GS_00096 | G | 1 | 6 | 16 | 0 |  | Y | Y | - |  | - | - | - | - | - | 6 | - | - | - | - | - | - | - |
| GS_21785 | - | 0 | 0 | 0 | 0 |  | - | Y | - |  | - | - | - | - | - | - | 4 | - | - | - | - | - | - |
| GS_05746 | M01 | 8 | 9 | 47 | 0 |  | Y | - | - |  | - | - | - | - | - | - | - | 4 | - | - | - | - | - |
| GS_23920 | Un | 2 | 2 | 5 | 0 |  | - | Y | - |  | - | - | - | - | - | - | - | - | 5 | - | - | - | - |
| GS_19074 | - | 0 | 1 | 10 | 0 |  | Y | - | - |  | - | - | - | - | - | - | - | - | - | 3 | - | - | - |
| GS_19777 | G | 5 | 18 | 50 | 0 |  | - | Y | Y |  | - | - | - | - | - | - | - | - | - | 17 | 14 | - | - |
| GS_01900 | - | 0 | 24 | 59 | 0 |  | - | Y | - |  | - | - | - | - | - | - | - | - | - | - | 5 | 3 | - |
| GS_08951 | - | 7 | 14 | 54 | 0 |  | Y | - | - |  | - | - | - | - | - | - | - | - | - | - | 9 | - | - |
| GS_19445 | A01A | 1 | 4 | 7 | 0 |  | Y | - | - |  | - | - | - | - | - | - | - | - | - | - | - | 18 | - |
| GS_14901 | A01A | 1 | 5 | 7 | 0 |  | Y | - | - |  | - | - | - | - | - | - | - | - | - | - | - | 4 | - |
| GS_21295 | - | 10 | 17 | 30 | 3 |  | - | Y | - |  | - | - | - | - | - | - | - | - | - | - | - | 16 | - |
| GS_08343 |  | 60 | 32 | 13 | 14 |  | Y | - | - |  | - | - | - | - | - | - | - | - | - | - | - | - | 16 |
| GS_12574 | A01A | 0 | 0 | 8 | 0 |  | Y | - | - |  | - | - | - | - | - | - | - | - | - | - | - | - | 4 |
| GS_15316 | A01A | 3 | 2 | 8 | 0 |  | Y | - | - |  | - | - | - | - | - | - | - | - | - | - | - | - | 4 |
| ^1^Protein designation from translated protein sequences ([17](#_ENREF_17)). ^2^Peptidase family as defined in the MEROPS data base ([22](#_ENREF_22)), G refers to O-glycosyl hydrolase, Un refers to unclassified peptidase, - refers to annotation of another function; see more complete annotation in Table S1. ^3^Fractions in addition to the Concanavalin A fraction in which proteins were identified (numbers refer to quantity of mass spectra): PBS or 4MU, PBS or 4 M urea intestinal perfusates, respectively; P2, 5K-50K x *g* intestinal pellet; Pf, pseudocoelomic fluid. ^4^SP, signal peptide; NC, Non-classical secretion; TM, transmembrane. ^5^Calculated mass for gel slice from SDS-PAGE gel, obtained as described in Methods, the numbers refer to quantity of mass spectra obtained for each protein. | | | | | | | | | | | | | | | | | | | | | | | |
