# Supplementary material for: Peptidases Compartmentalized to the Ascaris suum Intestinal Lumen and Apical Intestinal Membrane
Source: PLoS Negl Trop Dis. 2015 Jan 8;9(1):e3375. doi: 10.1371/journal.pntd.0003375 (PMC4287503; doi:10.1371/journal.pntd.0003375)
Supplement: S1 Text — Detailed materials, methods and analysis. (PDF) [file pntd.0003375.s004.pdf]

Supporting Information S1: Detailed materials, methods and analysis

Methods

Parasite material and intestinal fractionation. Adult female *Ascaris suum* were obtained from swine infected as weanling pigs (mixed breed, Swine Center, Washington State University) with 50 *A. suum* eggs containing infective larvae each of six times over a two week period. Swine were maintained in a vivarium under standard care for 60 to 70 days post-infection, after which they were euthanized and necropsied to obtain adult worms from the small intestine. All animal protocols utilized here were approved by the Institutional Animal Care and Use Committee, Washington State University. Intestinal samples were dissected from isolated worms maintained in ice cold phosphate buffered saline (PBS, pH 7.4). Worms used for cannulation were maintained in PBS at 37^0C until processed for cannulation, which was done at room temperature.

Protein fractions. Intestine from Adult female *A. suum* was dissected and stored at -80^0C. To generate protein samples for peptidase experiments, samples were ground in liquid nitrogen using a mortar and pestle. Fifty microliters of ground frozen sample were transferred to a microfuge tube and 1ml of PBS was added to the sample which was briefly vortexed and then treated in 3 rounds of a freeze (-20^0C) thaw (4^0C) cycle. The homogenate was then centrifuged at 5,000 x *g* (5kg) for 10 minutes, which produced a 5kg pellet (P1) and supernatant (S1). The S1 supernatant was centrifuged at 50,000 x *g* (50kg) for 30 minutes producing a 5kg to 50kg pellet (P2) and supernatant (S2). PBS and 4MU intestinal perfusates were prepared from cannulated worms as described above. PF was obtained by opening the body wall of adult female worms and collecting fluid that was released from the pseudocoelom. Protein concentrations were determined using a bicinchoninic acid assay (Micro BCA™ Protein Assay Kit, Thermo Scientific, Rockford, IL).

Cannulation and collection of intestinal perfusates. The anterior end of adult female *A. suum* worms was removed by scalpel just below the esophagus. A blunt needle cannula (25 guage) was inserted into the anterior end of the intestine, and the worm body was attached with superglue gel onto the cannula. The posterior one sixth of the worm was removed to eliminate a fragile section of the intestine. Approximately one centimeter of the remaining posterior end of the intestine was exposed by dissecting away the body. This step allowed collection of luminal contents by perfusion from the intestine with minimal contamination of fluid that emanated from the pseudocoelom. Perfusion with dye indicated a lumenal content of ca. 50 *u*l. Cannulated intestines were perfused with approximately 300 to 500 *u*l of PBS (PBS perfusate), followed by a similar volume of 4 M urea in PBS (4MU perfusate), each delivered with a tuberculin syringe attached to the cannula. Perfusates were collected on Parafilm M® (Pechiney Plastic Packaging Co., Chicago, IL), which allowed manipulation of the posterior end of the intestine in positions to reduce contamination of pseudocoelomic fluids (PFs). Maintenance of worms at 37^0C prior to cannulation appeared to enhance the flow of perfusates through the intestine. After collection, perfusate samples were stored at -20^0C until used for peptidase assays and analysis by mass spectrometry.

Concanavalin A (ConA) binding proteins. Whole intestinal lysate was prepared with peptidase inhibitors (1µM Pepstatin A, 1 mM PMSF, 10 mM 1,10 Phenanthroline, 5mM Iodoacetamide, Sigma, St. Louis, MO) and solubilized with 1% sodium dodecyl sulfate (SDS). The lysate was clarified by centrifugation at 5,000 x g and then the supernatant was diluted to 0.25% SDS with Binding Buffer containing divalent cations (50 mM TRIS [pH 7.4], 500 mM NaCl, 1mM each $MgCl_2$, $MnCl_2$, and $CaCl_2$). ConA-agarose beads (Sigma, St. Louis, MO) were incubated with intestinal lysate (2 mg per 200µl packed beads) The mixture was incubated with

inversion for 2 hours, and then washed 3 times with 9 bead volumes of Binding Buffer (50 mM TRIS, pH 7.4, 500 mM NaCl). Sequential 300 µl washes of potassium thiocyanate (0.25M, 0.5M, 1M and 2 M, in 20 mM TRIS [pH 7.4], 0.2% Triton X-100, two washes per concentration) were followed by two Binding Buffer washes (9 bead volumes each). Proteins remaining on the beads were eluted by boiling in 0.1% SDS for separation by SDS-polyacrylamide gel electrophoresis (PAGE) and mass spectrometric analysis.

Histological analysis. 10% formalin fixed adult female worms embedded in paraffin (Histology Laboratory, Washington State University) were sectioned attached to glass slides and deparaffinized and steam treated, To assess specificity of ConA binding, sections were treated with sodium periodate (5mM in 50 mM sodium acetate buffer, pH 4.5), followed by sodium borohydride (50 mM in PBS, pH 7.4) to disrupt carbohydrates containing vicinyl hydroxyls. Slides were ten treated with 0.3% hydrogen peroxide in methanol for 30 min at 25°C to eliminate endogenous peroxidases and then incubated with ConA-horse radish peroxidase (HRPO). Binding was localized by development with Metal Enhanced DAB Substrate (Thermo Scientific, Rockford, IL.). Sections were then counterstained with Mayer's haematoxylin (Thermo Scientific, Fremont, CA).

SDS-PAGE analysis. Samples were separated by SDS-PAGE under non-reducing conditions, using 17% to 7% polyacrylamide gradient gels using previously described methods (15). Molecular weight standards (PageRuler™ Plus Prestained Protein Ladder, Thermo Scientific, Rockford , IL) were used to estimate M*r* of separated proteins that were either stained with Coomassie Brilliant Blue R-250 (BioRad, Hercules, CA) for visualization, or transferred to nitrocellulose for probing with Concanavalin A-horse radish peroxidase (Con A-HRPO) conjugate (L6397, Sigma, St. Louis, MO). Filters were then incubated with Con A-HRPO (2 µg/ml in PBS [pH 7.4], 0.05% Tween 20, 1 mM each $CaCl_2$, $MnCl_2$ and $MgCl_2$) for 4 hrs, followed by 3 x 10 min rinses in 100 ml PBS (pH 7.4), 0.05% Tween 20. Localization of Con A-HRPO was visualized by chemiluminescence (Pierce® ECL Western Blotting Substrate, Thermo Scientific, Rockford IL) and recorded on x-ray film (Kodak™O-MAT™). To assess specificity of ConA binding, replicate nitrocellulose filters were treated with sodium periodate (5mM in 50 mM sodium acetate buffer, pH 4.5), followed by sodium borohydride (50 mM in PBS, pH 7.4) to disrupt carbohydrates containing vicinyl hydroxyls. Alternatively, control filters were treated identically, but the sodium periodate treatment was excluded.

Peptidase assays. For analysis of soluble protein, samples were adjusted to 1% TX-100 and used in assays at 1 to 4 ugs (depending on assay) per well. Buffers used were 100 mM citrate phosphate (pH 5.0-7.0) and 100 mM phosphate (pH 8.0) and incubated with Bodipy FL™ casein (E6638, Life Technologies, Grand Island, NY) for 2 hours in a C-1000 Touch™ thermal cycler with a CFX96™ Optical Reaction Module (Bio-Rad, Hercules, CA) at 37°C, in a total volume of 50 µl. Assays were conducted in triplicate using 96 well PCR plates (Bio-Rad, Hercules, CA). Fluorescence generated during digestion was measured (excitation 490 nm; emission 530 nm). Net fluorescence signal was determined by subtraction of starting values from end values. Mean fluorescence (three wells) was calculated for no protein samples after incubation with Bodipy FL™ casein at a given pH for two hrs. This background value was subtracted from each fluorescence value obtained for samples with protein. Activity was expressed as relative fluorescence units per ug of protein.

For analysis of ConA binding proteins, intestinal supernatant S1 solubilized in 1% TX-100 was incubated with beads (1 mg per 100 µl packed beads) for 2 hours with inversion, then washed with Binding Buffer containing divalent cations (50 mM TRIS, [pH 7.4], 500 mM NaCl, 1mM each $MgCl_2$, $MnCl_2$, and $CaCl_2$). Con A-agarose beads with bound proteins (10 µl) were transferred to wells of 96 well flat-bottomed plates (Corning Costar®, Corning, NY) in 50 µl total

volume for assays as described for peptidase assays. Beads with no proteins were used for no protein controls. The 96 well plates were rotated during incubation (50 rpm) to ensure mixing. Reaction supernatants from which beads were eliminated were transferred to a 96-well PCR plate (Bio-Rad, Hercules, CA) for fluorimetric measurements, as described for peptidase assays. Alternatively, SDS (1.0%) solubilized S1 supernatants were used for ConA bead isolation and analysis by mass spectrometry and testing in peptidase assays, but produced no detectable activity.

Statistical analysis. Mean fluorescence units generated from treatments in peptidase assays were compared first by analysis of variance (ANOVA), followed by Tukey's multiple comparison of means. ANOVA was conducted among treatment groups for individual pHs. For inhibition experiments, ANOVA and multiple means comparisons were conducted to identify means of inhibitor treated groups that differed from the untreated control group at a given pH. For significance with ConA-bead isolated proteins, mean fluorescence at each pH tested was assessed by 95% confidence intervals (CI) to determine if the CI was above zero.

Mass spectrometry – sample preparation. ConA bead-isolated proteins were separated on SDS-PAGE gels in two lanes, one of which was Coomassie Blue stained and the second was transferred to nitrocellulose and probed with Con A-HRPO, as described under SDS-PAGE analyisis. Bands detected in blots were used to align with stained bands in the gel, which were excised and then prepared for *in situ* trypsin digestion and analysis by LC-MS/MS as described [1]. Proteins in PBS and 4MU perfusates, PF, and P2 pellets were were precipitated with the "2-D Clean-up kit" (GE Healthcare Life Sciences), and then resolubilized in 8M urea, 100mM Tris, pH 8.5 (20ul) for 30min at 37ºC (with agitation if a pellet is observed). Sample were then reduced with 1mM tris(2-carboxyethyl)phosphine TCEP (2ul of 10mM TCEP stock) at room temperature for 30 minutes, followed by alkylation with 20mM Iodoacetamide for 30 minutes in the dark. Then, they were quenched with 10mM DTT for 15 minutes and 1:4 dilution in Tris (pH 8.5).

Liquid Chromatography, Tandem Mass Spectrometry (LC-MS/MS) Proteins were digested sequentially with endoprotease Lys-C (cleaving lysine at the C-termius) and trypsin as previously described [2]. A solid phase extraction of peptides with microtips of C4 and porous graphite carbon (GlyGen) was performed on a Bravo automated liquid-handling robot prior to LC-MS. Peptide mixtures were analyzed using high-resolution nano-LC-MS on a hybrid mass spectrometer consisting of a linear quadrupole ion-trap and an Orbitrap (Linear Trap Quadrupole[LTQ]-Orbitrap Elite, Thermo Fisher Scientific). Chromatographic separations were performed using a nanoLC 1D Plus™ (Eksigent) for gradient delivery and a cHiPLC-nanoflex (Eksigent) equipped with a 15 cm x 75 µm C18 column (ChromXP C18-CL, 3 µm, 120 Å, Eksigent). The liquid chromatograph was interfaced to the mass spectrometer with a nanospray source (PicoView PV550; New Objective). Mobile phases were 1% formic acid (FA) in water (A) and 1% FA in 99% acetonitrile (ACN; B). After equilibrating the column in 98% solvent A (aqueous 1% FA) and 2% solvent B (ACN containing 1% FA), the samples (10 µL) were injected from autosampler vials using the LC-system's autosampler at a flow rate of 750 nL/min followed by gradient elution (400 nL/min) with solvent B: isocratic at 2% B, 0-5 min; 2% B to 40% B, 5-135 min; 40% to 80%, 135-152 min; 80% to 2%, 152-155; and isocratic at 2% B, 155-170 min. Total run time, including column equilibration, sample loading, and analysis was 217 min. The survey scans (m/z 350-2000) (MS1) were acquired at high resolution (60,000 at m/z = 400) in the Orbitrap in profile mode and the MS/MS spectra (MS2) were acquired at 7500 resolution in the Orbitrap after fragmentation in the linear ion trap. The maximum injection times for the MS1 scan in the Orbitrap and the LTQ were both 500 ms, and the maximum injection times for the multi-level fragmentation scan (MS^n) in the Orbitrap and the LTQ were 800 ms and

5000 ms, respectively. The automatic gain control targets for the Orbitrap and the LTQ were 5 x 10^5 and 3 x 10^4, respectively for the MS1 scans and 2 x 10^5 and 1 x 10^4, respectively for the MS2 scans. The MS1 scans were followed by three MS2 events in the linear ion trap with collision activation in the ion trap (parent threshold = 10,000; isolation width = 4.0 Da; normalized collision energy = 30%; activation Q = 0.250; activation time = 30 ms). Dynamic exclusion was used to remove selected precursor ions (-0.20/+1.0 Da) for 90 s after MS2 acquisition. A repeat count of 3, a repeat duration of 45 s, and a maximum exclusion list size of 500 was used. The following ion source parameters were used: capillary temperature 200 ºC, source voltage 2.7 kV, source current 100 μA, and the tube lens at 79 V. The data were acquired using Xcalibur, version 2.0.7 (Thermo Fisher).

Data Processing and Analysis LC-MS data files (MS2 centroided) were used for database searching with MASCOT (Matrix Science, version 2.3.0.0) using previously described software settings [3], against the deduced *A. suum* proteome [4] and the *Sus scrofa* proteome (Uniprot, downloaded Sept. 2012) to identify potential contamination. The search was conducted with tryptic enzyme specificity, allowing 2 missed cleavages, oxidation of methionine (Met), carbamidomethylation of cysteine (Cys) as modifications. A parent ion tolerance of 20 ppm and a fragment ion mass tolerance of 100 absolute milli-mass units was used for all searches. Scaffold (version Scaffold_3.6.4, Proteome Software Inc., Portland, OR) was used to validate MS/MS based peptide and protein identifications [5]. Peptide identifications were accepted if they could be established at greater than 95.0% probability as specified by the Peptide Prophet algorithm [3]. Protein probabilities were assigned by the Protein Prophet algorithm, and protein identifications were accepted if they could be established at greater than 95.0% probability, with an overall 0.1% false discovery rate (FDR) at the protein level. These thresholds were set in accordance to the suggestions by the Scaffold documentation, and as described in other recent proteomics studies [6,7]. Proteins that contained similar peptides and could not be differentiated based on MS/MS analysis alone were grouped to satisfy the principles of parsimony.

References

1. Jasmer DP, Lahmers KK, Brown WC (2007) Haemonchus contortus intestine: a prominent source of mucosal antigens. Parasite Immunol 29: 139-151.
2. Chen Z-W, Fuchs K, Sieghart W, Townsend RR, Evers AS (2012) Deep Amino Acid Sequencing of Native Brain GABAA Receptors Using High-Resolution Mass Spectrometry. Molecular & Cellular Proteomics 11.
3. Nesvizhskii AI, Keller A, Kolker E, Aebersold R (2003) A statistical model for identifying proteins by tandem mass spectrometry. Anal Chem 75: 4646-4658.
4. Jex AR, Liu S, Li B, Young ND, Hall RS, et al. (2011) Ascaris suum draft genome. Nature 479: 529-533.
5. Keller A, Nesvizhskii AI, Kolker E, Aebersold R (2002) Empirical statistical model to estimate the accuracy of peptide identifications made by MS/MS and database search. Anal Chem 74: 5383-5392.
6. Biesemann C, Grønborg M, Luquet E, Wichert SP, Bernard V, et al. (2014) Proteomic screening of glutamatergic mouse brain synaptosomes isolated by fluorescence activated sorting. The EMBO Journal 33: 157-170.
7. Connolly GC, Hagen F, Willard B, Iyer R, McCrae KR (2013) A Proteomics-Based Approach To Identifying Mechanisms Of Cancer-Associated Thrombosis: Potential Role For Immunoglobulins. Blood 122: 1127.
