# Supplementary material for: Peptidases Compartmentalized to the Ascaris suum Intestinal Lumen and Apical Intestinal Membrane
Source: PLoS Negl Trop Dis. 2015 Jan 8;9(1):e3375. doi: 10.1371/journal.pntd.0003375 (PMC4287503; doi:10.1371/journal.pntd.0003375)
Supplement: Supplementary file 4 [file pntd.0003375.s001.docx]

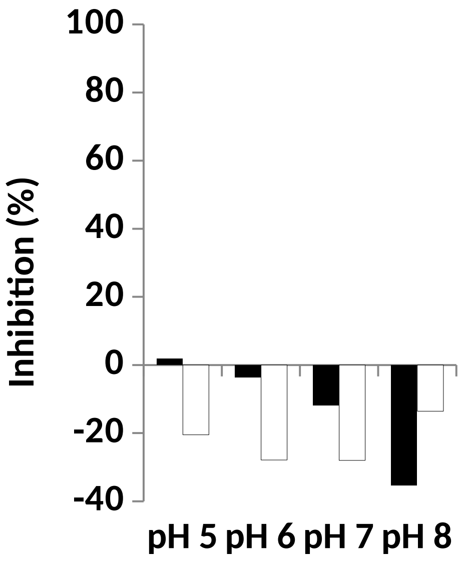

100
80
60
40
20
0
-20
-40
Inhibition (%)
pH 5
pH 6
pH 7
pH 8
